# HIV Care Coordination promotes care re-engagement and viral suppression among people who have been out of HIV medical care: an observational effectiveness study using a surveillance-based contemporaneous comparison group

**DOI:** 10.1186/s12981-021-00398-0

**Published:** 2021-10-12

**Authors:** Mary K. Irvine, McKaylee M. Robertson, Denis Nash, Sarah G. Kulkarni, Sarah L. Braunstein, Bruce Levin

**Affiliations:** 1 Bureau of Hepatitis, HIV and Sexually Transmitted Infections , New York City Department of Health and Mental Hygiene, 42-09 28th Street, CN 21-74, Queens, New York, NY 11101-4132 USA; 2 Institute for Implementation Science in Population Health (ISPH), Graduate School of Public Health and Health Policy, City University of New York (CUNY), New York, NY USA; 3 Department of Biostatistics, Mailman School of Public Health, Columbia University, New York, NY USA

**Keywords:** HIV care continuum, Cohort studies, Viral suppression, Care re-engagement, HIV surveillance, Case management, Ryan White, Public health, North America

## Abstract

**Background:**

Medical care re-engagement is critical to suppressing viral load and preventing HIV transmission, morbidity and mortality, yet few rigorous intervention studies address this outcome. We assessed the effectiveness of a Ryan White Part A-funded HIV Care Coordination Program relative to ‘usual care,’ for short-term care re-engagement and viral suppression among people without recent HIV medical care.

**Methods:**

The Care Coordination Program was launched in 2009 at 28 hospitals, health centers, and community-based organizations in New York City. Designed for people with HIV (PWH) experiencing or at risk for poor HIV outcomes, the Care Coordination Program provides long-term, comprehensive medical case management utilizing interdisciplinary teams, structured health education and patient navigation. The intervention was implemented as a safety-net services program, without a designated comparison group. To evaluate it retrospectively, we created an observational, matched cohort of clients and controls. Using the HIV surveillance registry, we identified individuals meeting program eligibility criteria from December 1, 2009 to March 31, 2013 and excluded those dying prior to 12 months of follow-up. We then matched clients to controls on baseline status (lacking evidence of viral suppression, consistently suppressed, inconsistently suppressed, or newly diagnosed in the past 12 months), start of follow-up and propensity score. For this analysis, we limited to those out of care at baseline (defined as having *no viral load* test in the 12 months pre-enrollment) and still residing within jurisdiction (defined as having a viral load or CD4 test reported to local surveillance and dated within the 12-month follow-up period). Using a GEE model with binary error distribution and logit link, we compared odds of care re-engagement (defined as having ≥ 2 laboratory events ≥ 90 days apart) and viral suppression (defined as having HIV RNA ≤ 200 copies/mL on the most recent viral load test) at 12-month follow-up.

**Results:**

Among 326 individuals out of care at baseline, 87.2% of clients and 48.2% of controls achieved care re-engagement (Odds Ratio: 4.53; 95%CI 2.66, 7.71); 58.3% of clients and 49.3% of controls achieved viral suppression (Odds Ratio: 2.05; 95%CI 1.30, 3.23).

**Conclusions:**

HIV Care Coordination shows evidence of effectiveness for care and treatment re-engagement.

## Introduction

The individual and population-level benefits of antiretroviral therapy (ART) for HIV depend upon consistent medical care to achieve and maintain viral suppression (VS) [1–3]. According to the Centers for Disease Control and Prevention (CDC) Compendium of Evidence-Based Interventions and Best Practices for HIV Prevention, multiple interventions show strong evidence of efficacy for initial linkage to care, subsequent retention in care and VS, but *none have generated strong evidence of efficacy for care re-engagement (CR) following a lapse* [4]. Promising CR approaches include case management, patient navigation, outreach and uses of population-based data or routine testing to identify candidates for re-linkage [5–14]. However, studies to date have lacked contemporaneous, comparable out-of-care control groups [7–15] or have focused on linkage and retention rather than quantifying CR [5–7].

In December 2009, the New York City (NYC) Health Department launched a Ryan White Part A—funded comprehensive medical case management intervention known as the HIV Care Coordination Program (CCP). The CCP has demonstrated effectiveness for VS and for durable VS (defined as regular monitoring and all viral loads ≤ 200 copies/mL in months 13–36 of follow-up) among previously unsuppressed individuals [16–18], but it has not been examined for its effect on CR. The objective of this analysis was to assess CCP versus usual-care effectiveness for CR and VS among people with HIV (PWH) lacking recent HIV medical care. We hypothesized that the CCP would show CR and VS benefits over and above usual care for this subgroup of PWH.

## Methods

### Intervention

The CCP employs a ‘medical home’ model combining interdisciplinary team-based case management, patient navigation and structured health education to promote HIV care continuum engagement. Its components and implementation considerations are described elsewhere [7, 19, 20], and a toolkit for replication is online [21]. During the period analyzed, CCP protocols permitted enrollment of HIV-positive adults or emancipated minors who were eligible for local Ryan White Part A services (living at < 435% of federal poverty level and within the New York grant area) *and* (a) newly diagnosed; (b) never in care or out of care for at least nine months; (c) irregularly in care; (d) starting a new ART regimen; (e) experiencing ART adherence barriers; or (f) manifesting treatment failure or ART resistance [7].

### Data sources

The NYC HIV Surveillance Registry (“the Registry”) contains demographic information and comprehensive HIV-related laboratory reporting [including all CD4 and viral load (VL) results] for individuals with NYC HIV medical care. Vital status is updated through regular matches with death data [22]. Ryan White Part A programmatic data and Registry data are matched semi-annually for merged analysis.

Using the merged dataset, we identified people enrolled in the CCP from December 1, 2009 to March 31, 2013 and excluded clients dying within 12 months post-enrollment [N (number) = 279]. We then identified unenrolled individuals diagnosed with HIV by March 31, 2013 and ≥ 18 years old at diagnosis. All demographic, baseline, outcome and death data were drawn from the Registry.

### Comparison group construction

Via a four-step process detailed elsewhere [23] and summarized below, we retrospectively created an observational, matched cohort of CCP and non-CCP PWH.

First, we identified *CCP eligibility windows* for unenrolled PWH: ranges of time between December 2009 and March 2013 during which they appeared CCP-eligible based on laboratory test data from the Registry [23]. We considered PWH eligible if they were (1) *newly diagnosed*; (2) *out of medical care at least 9 months*; (3) *treatment naïve* [24]; (4) *lacking VS or lacking VL tests* in the 12 months after ART initiation [24]; (5) *experiencing viral rebound* following VS; or (6) *registering a high VL* (≥ 10,000 copies/mL). To ensure comparability with the CCP group, we closed eligibility windows ≥ 12 months prior to any date of death.

Second, from within their eligibility window(s), we randomly assigned each non-CCP individual a pseudo-enrollment date (time point from which to start follow-up). Pseudo-enrollment dates were assigned with probabilities such that their temporal distribution matched that of the CCP clients’ enrollment dates.

Third, we restricted to people with at least one CD4 or VL in the 24 months post-enrollment/pseudo-enrollment. We required one laboratory test as a proxy for ongoing receipt of NYC medical care, to prevent a differential (non-CCP versus CCP) effect of outmigration.

Finally, we matched CCP enrollees to eligible non-CCP PWH on baseline treatment status, enrollment/pseudo-enrollment date, and propensity for CCP enrollment. Correctly specified propensity models balance measured confounders across exposure groups [25]. We estimated the propensity score by modeling exposure status as a function of the confounders of the relationship between exposure and outcome. To begin, we developed an a priori list of variables considered to be potential confounders of the relationship between enrollment in the CCP and the outcome of VS: sex, race/ethnicity, age at enrollment/pseudo-enrollment, country of birth, HIV transmission risk, year of diagnosis, baseline VL, baseline CD4, successful linkage to HIV care within three months of diagnosis, presence of an AIDS diagnosis within one year of HIV diagnosis, number of VL laboratory tests reported in the year prior to enrollment/pseudo-enrollment, residential Zone Improvement Plan (ZIP) code at enrollment/pseudo-enrollment, HIV prevalence and poverty level within ZIP code at enrollment/pseudo-enrollment and interaction terms for baseline CD4 and baseline VL, baseline CD4 and race, sex and risk, and year of diagnosis and risk [23].

Baseline treatment status was defined in terms of VS or diagnosis in the 12 months pre-enrollment/pseudo-enrollment: (1) ‘lacking evidence of VS’ (no VL ≤ 200 copies/mL), (2) ‘consistently suppressed’ (at least two VLs ≥ 90 days apart and all VLs ≤ 200 copies/mL), (3) ‘inconsistently suppressed’ (at least one VL ≤ 200 copies/mL, but not all VLs ≤ 200 copies/mL), or (4) ‘newly diagnosed.’ We used logistic regression to estimate propensity for CCP enrollment within baseline treatment status groups, starting with a model that included all a priori confounders and applying backward selection to identify the model with the lowest value of Akaike’s Information Criterion (AIC) [23]. Within baseline treatment status groups, we matched on propensity scores and enrollment/pseudo-enrollment dates (within three months), using a 1:1 greedy match algorithm that proceeded sequentially from 8 to 1 decimal places of the propensity score [26, 27]. The final model and match was chosen based on having no between-group imbalance (standardized difference ≥ 0.1) in any measured confounder and the greatest number of persons matched [25]. In a previously published sensitivity analysis, we ran models using all hypothesized confounders; the effect estimates did not differ from the approach described above; however, fewer CCP enrollees were matched [23].

### Definitions

#### Out of care and residing in NYC

To preserve the original match to the extent possible, we defined ‘out of care’ as a subcategory of the ‘lacking evidence of VS’ treatment status group: those with *no VL* reported in the year before enrollment/pseudo-enrollment. Any Registry-reported CD4 or VL test in the *first 12 months* of follow-up was considered evidence of NYC residency. The post-hoc requirement of a CD4/VL in the *first 12 months* versus the *first 24 months* (third step, above) was applied to align with the 12-month CR/VS outcome timeframe.

#### Outcomes

CR was dichotomized as ≥ 2 laboratory events (CD4 or VL) ≥ 90 days apart in the 12-month period post-enrollment/pseudo-enrollment. VS was dichotomized as a value ≤ 200 copies/mL on the last VL in that period.

### Study population and period

From December 1, 2009 to March 31, 2013, 7,337 PWH enrolled in the CCP; 7,058 (96.2%) were living 12 months post-enrollment. Of the 62,828 unenrolled CCP-eligible PWH, 91.9% (57,746) were assigned a pseudo-enrollment date; 74.8% (46,997) had an HIV-related NYC laboratory test in the 24 months following their pseudo-enrollment date; and 10.8% (6,812) were matched to a CCP client, resulting in 6,812/7,058 CCP clients matched (96.5%). Of 5,666 PWH ‘lacking evidence of VS’ at baseline in the matched cohort, 326 were ‘out of care and residing in NYC’: 148 non-CCP and 178 CCP PWH. In all, the records used for this study spanned the period from December 1, 2007 through March 31, 2015. The laboratory observation period started 24 months earlier than the enrollment period because CCP eligibility was based on clinical status in the past 24 months, and it extended 24 months past the end of the enrollment period because the match was restricted to PWH who had an HIV-related NYC laboratory test in the 24 months following enrollment/pseudo-enrollment.

### Statistical analysis

In an intention-to-treat approach, we used a generalized estimating equation (GEE) model with binary error distribution and logit link to estimate the CCP versus non-CCP odds ratio (OR) for CR and for VS, accounting for the matched pair design by specifying the pairs as the independent clusters in the GEE model with an exchangeable working correlation structure. The model included three terms: CCP participation (yes or no), out-of-care status at baseline (yes or no) and an interaction term for CCP participation and care status. The CCP effect within the out-of-care group (N = 326) was generated from the interaction term from the entire cohort (N = 13,624) to account for propensity matching and balanced covariates [25, 28]. ORs were estimated with GENMOD procedure in SAS version 9.5.

### Human subjects

This study was approved by the institutional review boards at the NYC Department of Health and Mental Hygiene and the City University of New York (CUNY) Graduate School for Public Health and Health Policy. For these retrospective secondary analyses of de-identified data, we received a waiver of informed consent in accordance with the pre-2018 requirements in 45 CFR (Code of Federal Regulations) 46.116(d)(2).

## Results

The out-of-care CCP and non-CCP groups were similar on race, age and country of birth (Table 1). Overall (N = 326), 50% were Black, 40% Hispanic/Latinx and 6% White; 70% were United States (US)-born; and 57% were under age 45. The CCP group had a higher proportion of men (71% versus 62%) and men who have sex with men (43% versus 28%) than the non-CCP group. As expected, most CCP and non-CCP PWH with no VL also lacked a CD4 count in the pre-enrollment/pseudo-enrollment year (68% and 56%, respectively).

**Table 1:** Characteristics of clients and matched controls who had been out of care at baseline

	Total		Non-CCP		CCP	
	N	%	N	%	N	%
Total	326	100.0	148	100.0	178	100.0
Male	217	66.6	91	61.5	126	70.8
Female	109	33.4	57	38.5	52	29.2
Race/ethnicity						
Black	162	49.7	74	50.0	88	49.4
Hispanic/Latino(a)	129	39.6	58	39.2	71	39.9
White	21	6.4	8	5.4	13	7.3
Other	14	4.3	8	5.4	6	3.4
Age category at baseline						
18–24	17	5.2	6	4.1	11	6.2
25–44	169	51.8	75	50.7	94	52.8
45–64	133	40.8	63	42.6	70	39.3
65+	7	2.1	4	2.7	3	1.7
Transmission risk						
Men who have sex with men	118	36.2	42	28.4	76	42.7
Injection drug use history	60	18.4	30	20.3	30	16.9
Heterosexual	77	23.6	37	25.0	40	22.5
Other/unknown	71	21.8	39	26.4	32	18.0
Country of birth						
US/US Territory	227	69.6	106	71.6	121	68.0
Foreign Born	56	17.2	27	18.2	29	16.3
Unknown	43	13.2	15	10.1	28	15.7
Year of HIV diagnosis						
Prior to 1995	44	13.5	19	12.8	25	14.0
1995–1999	47	14.4	23	15.5	24	13.5
2000–2004	100	30.7	37	25.0	63	35.4
2005–2009	116	35.6	58	39.2	58	32.6
2010–2013	19	5.8	11	7.4	8	4.5
Baseline CD4 count						
< 200	44	13.5	21	14.2	23	12.9
200–349	29	8.9	16	10.8	13	7.3
350–499	20	6.1	12	8.1	8	4.5
500+	29	8.9	16	10.8	13	7.3
Missing	204	62.6	83	56.1	121	68.0
HIV prevalence and poverty level in ZIP code of residence at baseline						
High poverty and prevalence	200	61.3	93	62.8	107	60.1
Low poverty and high prevalence	56	17.2	20	13.5	36	20.2
High poverty and low prevalence	21	6.4	8	5.4	13	7.3
Low poverty and prevalence	31	9.5	17	11.5	14	7.9
Unknown	18	5.5	10	6.8	8	4.5

*CCP* Care Coordination Program, *N* number, *US* United States, *ZIP* Zone Improvement Plan

CCP clients had significantly greater odds of CR and VS at 12-month follow-up (Table 2). The proportion of out-of-care NYC residents re-engaged in care was 88% in the CCP versus 63% in the usual-care group (OR: 4.53; 95% confidence interval [CI] 2.66, 7.71). The proportion achieving VS was 66% in the CCP versus 49% in the usual-care group (OR: 2.05; 95% CI 1.30, 3.23).

**Table 2:** Odds ratios for care re-engagement and viral suppression, among individuals out of care at baseline

	Denominator	Numerator	(%)	OR	(95% CI)
Care re-engagement					
Total	326	250	(76.69)		
CCP	178	157	(88.20)	4.53	(2.66, 7.71)
Non-CCP (Ref)	148	93	(62.84)		
Viral suppression					
Total	326	190	(58.28)		
CCP	178	117	(65.73)	2.05	(1.30, 3.23)
Non-CCP (Ref)	148	73	(49.32)		

*CCP* Care Coordination Program, *Ref* reference category, *OR* odds ratio, *CI* confidence interval

## Discussion

### Summary and context

Out-of-care CCP enrollees had four and a half times the odds of CR and twice the odds of VS at 12-month follow-up, compared to similar but unenrolled out-of-care PWH. These findings have implications for programming/policy efforts to end the epidemic, as the CDC estimates that the greatest share of HIV transmission events (43%) involve people aware of their HIV status but not in HIV care [29]. As a rigorously evaluated intervention demonstrating substantial, real-world effectiveness at re-engaging PWH who have been out of HIV care, the CCP could be deployed more broadly for the purpose of reducing delays or interruptions in HIV care, thus reducing HIV transmission and improving both health and survival among PWH.

Recent reviews highlight the dearth of rigorous studies demonstrating intervention effects on CR or even assessing CR as an outcome [4, 30, 31]. A King County, Washington clinic-based study of a data-to-care intervention reported modest re-linkage benefits; compared to historical controls, intervention recipients were re-engaged more quickly (adjusted Hazard Ratio: 1.7 [95% CI 1.2, 2.3]) and more frequently (15% versus 10%, adjusted Relative Risk: 1.6 [1.2, 2.1]) [8]. However, as with other re-engagement studies lacking a contemporaneous comparison group [7, 9, 10, 12, 13, 15], estimates may have been affected by secular trends.

Previously, the CCP was found to improve 12-month VS relative to usual care [16]. Our current results extend those findings: the CCP promotes 12-month *CR and VS* for previously *out-of-care* PWH. Comparison with other case management intervention studies is complicated by their varying settings, designs, endpoints and populations. In a randomized trial of a case management-type intervention directed to new clients and clients with poor clinic attendance, greater retention was observed in the intervention versus usual-care group [5]. Another randomized trial showed that patient navigation-enhanced case management increased linkage and retention of PWH discharged from jail [6]. Our findings contribute further evidence of the HIV care continuum benefits of case management and patient navigation-enhanced case management, specifically, for PWH experiencing or at risk for gaps in care and treatment.

### Limitations and strengths

This study is subject to the limitations attending observational analyses, including potential uncontrolled confounding. Our reliance on Registry data enabled us to control for numerous demographic and clinical confounders [23], but not for behaviors or for services beyond the CCP. We also lacked direct data on out-migration from NYC. The restriction to PWH with at least one CD4 or VL in the 12 months post-enrollment/pseudo-enrollment was applied to avoid bias from differential out-migration between CCP and non-CCP PWH, and resulted in more conservative CCP effect estimates than the less restricted analysis (results not shown).

Strengths of our multi-site study included leveraging longitudinal outcome data from a single, comprehensive source for all NYC PWH, regardless of care location within NYC or CCP enrollment status. Availability of complete surveillance data on both outcomes supported an intention-to-treat analysis. Furthermore, use of a contemporaneous out-of-care comparison group matched to CCP enrollees on follow-up timing and propensity scores minimized the risk that observed effects could result from secular outcome improvements or group differences on measured confounding variables.

## Conclusions

Our findings fill a gap in the literature by providing *strong* evidence of one case management program’s effectiveness for *re-engaging* PWH in HIV care and treatment. As care engagement often does not follow a simple linear progression [32], re-engagement strategies are essential to preventing HIV transmission and HIV-related morbidity and mortality. Rigorous, real-world studies assessing effects on re-engagement can guide policymakers in selecting interventions to speed the end of the HIV epidemic.

## Data Availability

Materials on the Care Coordination intervention and its components are available at the CDC websites: https://www.cdc.gov/hiv/effective-interventions/treat/steps-to-care/index.html and https://www.cdc.gov/hiv/research/interventionresearch/compendium/lrc/completelist.html, as well as through the corresponding author. Due to legal restrictions (New York Public Health Law Article 21, Title III) and the confidential nature of HIV surveillance data in New York, public health authorities in New York City cannot release de-identified individual-level data on reported HIV cases for purposes other than ensuring appropriate HIV care. However, Health Department staff can provide code and assistance to external researchers with further specific data questions or uses, on reasonable request via e-mail to hivreport@health.nyc.gov.

## Abbreviations

ART: Antiretroviral therapy
VS: Viral suppression
CDC: Centers for Disease Control and Prevention
CR: Care re-engagement
NYC: New York City
CCP: Care Coordination Program
PWH: People with HIV
VL: Viral load
N: Number
ZIP: Zone Improvement Plan (United States Postal Service-defined geographic unit)
AIC: Akaike’s Information Criterion
GEE: General estimating equation
OR: Odds ratio
CUNY: City University of New York
CFR: Code of Federal Regulations (United States)
US: United States
CI: Confidence interval
NIMH: National Institute of Mental Health
HRSA: Health Services and Resources Administration
CHORDS: Costs, Health Outcomes and Real-world Determinants of Success (study name)
